# Self through the Mirror (Neurons) and Default Mode Network: What Neuroscientists Found and What Can Still be Found There

**DOI:** 10.3389/fnhum.2013.00383

**Published:** 2013-07-24

**Authors:** Stefano Sandrone

**Affiliations:** ^1^NATBRAINLAB – Neuroanatomy and Tractography Brain Laboratory, Department of Forensic and Neurodevelopmental Sciences, Institute of Psychiatry, King’s CollegeLondon, UK; ^2^Institute of Neuroinformatics, University of Zurich and ETH ZurichZurich, Switzerland

A search for the word *self* in the Stanford Encyclopedia of Philosophy finds 1187 entries: trying to give a complete definition of this concept is not so easy. It is a great puzzle that states who we are in the world, as Alice in Wonderland once argued, afraid she could not explain herself when she came across the Caterpillar. Unfortunately, we cannot use such an excuse. Furthermore, as neuroscientists, trying to depict the self from a scientific perspective seems to get harder and harder the deeper we get into our knowledge of the brain’s structure and functions. Generally speaking, the self has been seen through different lenses, according to the dominant *zeitgeist*. Classical cross-cultural studies confirmed what was intuitively conceivable: the concept of the self is highly varied across social groups and across traditions, mainly clustered around the well-characterized dichotomy between western and eastern visions (Markus and Kitayama, 1991; Baumeister and Finkel, 2010; Martínez Mateo et al., 2013), where the first is considered more independent and the latter more interconnected. In recent years, research on the self has significantly increased thanks to the cross-fertilization of disciplines that were once considered separated, such as philosophy, psychology, psychiatry, and neuroscience (Gallagher, 2011) and, to a certain extent, all the other neuro-related disciplines such as neuroethics, neuroesthetics, and neuroeconomics (Legrenzi and Umiltà, 2011). Old questions can now be addressed through recently developed – and still improving – technological tools falling under the term “neuroimaging,” such as positron emission tomography (PET) or functional magnetic resonance imaging (fMRI). As the self is by definition multifaceted and polyhedral both in space and time, the flourishing of new, twisted viewpoints is thus useful to further shape and deepen our knowledge on this intriguing topic. Remarkably, the investigation on the self benefits from two of the most recent discoveries, both of them claimed to be serendipitous, made in the highly interdisciplinary field of neuroscience: the Mirror Neuron System (MNS) and the Default Mode Network (DMN).

On the one side, the MNS mechanisms first unify execution and perception of an action, with a set of neurons, ranging from premotor and supplementary motor areas to primary somatosensory and inferior parietal cortices, coding for a precise action and activated also in the observers’ motor system (Cattaneo and Rizzolatti, 2009; Keysers et al., 2010). Although it has been sometimes misinterpreted (Rizzolatti and Sinigaglia, 2010), MNS is crucial for the study of the self. In fact, frontoparietal mirror neuron areas are crucial for the motor-simulation mechanisms, as well as cortical midline structures engaged in self-related information processing (Uddin et al., 2007) both in normal and pathological brain, as it can be seen for example in autistic (Enticott et al., 2012; Gallese et al., 2013) and schizophrenic subjects (Ferri et al., 2012; McCormick et al., 2012; Mehta et al., 2012).

On the other side, since 2001 it has been understood that when an individual is alerted though not actively engaged in cognitive tasks, spontaneously organized neural activity occurs in a unique constellation of brain regions called DMN and involving the posterior cingulate cortex, the precuneus, and regions of the ventromedial prefrontal cortex (Raichle et al., 2001; for an account of its discovery please refer to Raichle and Snyder, 2007; Buckner, 2012). The DMN has been consistently reported to be related to self-referential processing. In fact, activations and deactivations of DMN brain regions have often been related to self-specific processes in both healthy and diseased conditions (Gusnard et al., 2001; Sheline et al., 2009; Irish et al., 2012). Each area of the DMN seems to be involved in different subfunctions of self-referential processing (van Buuren et al., 2010), and a detailed map of the anatomo-functional DMN subregions is currently in progress (Salomon et al., 2013).

Even though some methodological caveats should be properly addressed and hopefully solved in the next years as far as resting states are concerned (Northoff et al., 2010, 2011), it is more and more evident that the boundaries between the perception of the “self” and the “other” should be pivotally found (also) around the CMS. Furthermore, being directly involved into both MNS and DMN, the self is at a crossroads between these two discoveries, and it is conceivable that in the near future such research on the self will be better valorized and further propelled through new insights that can directly derive from studying the MNS and DMN. The relationship between MNS and the self as well as its links with the study of the self and internal/external stimuli started to be discussed both for MNS (Sinigaglia and Rizzolatti, 2011) and DMN (Qin and Northoff, 2011), but a synergy between researchers from different subfields (of neuroscience and above) is strongly required to further look at the self through the mirror neurons and the DMN looking glass.

Remarkably, the first evidence of mirror neurons was obtained in monkeys with electrophysiological studies (di Pellegrino et al., 1992) and then replicated on man with neuroimaging techniques (Kilner et al., 2009; but see also Lingnau et al., 2009), electrophysiological recordings (Mukamel et al., 2010), and cerebral stimulation devices (Cattaneo et al., 2010; Avenanti and Urgesi, 2011). Instead, the opposite happened for the DMN. Experiments were carried out first on humans, then on chimpanzees (Rilling et al., 2007), monkeys (Kojima et al., 2009; Mantini et al., 2011) and, more recently, on rats (Lu et al., 2012), thus suggesting that DMN can be a crucial aspect of the mammalian brain. Replicating the data obtained from the “Mirror Test” (Gallup, 1970, 1994; Gallup et al., 2004) and investigating the emerging self, MNS, and DMN while performing the test or during the resting state may provide deep insight, and may help describe the emergence of the self from an evolutive perspective. Moreover, DMN activity has been longitudinally elucidated across the whole life cycle, namely from its emergence in 2-day-old newborns (Gao et al., 2009) to its disappearance in dead brain patients (Boly et al., 2009). Similarly, the ontogeny of social relationship has been addressed from twin fetuses (Castiello et al., 2010) onward (Kilner and Blakemore, 2007; Lepage and Théoret, 2007). Therefore, the developmental and maturational processes and the boundaries of the self can be further addressed with a combined MNS and DMN approach. In addition, both MNS and DMN seem to be altered in neurological and psychiatric conditions (Iacoboni and Dapretto, 2006; Sandrone, 2012, 2013), and, interestingly, abnormalities and disruptions recently started to be studied as predictive behavioral markers and clinical diagnostic tools. Future investigations will be aimed at capitalizing on clinical studies on neurological and psychiatric patients in order to improve the ability of DMN in discriminating single patients from single healthy controls with increasing sensitivity and high specificity, and if possible, to realize a joint MNS and DMN-based functional taxonomy of self-related diseases. Variations in the functional connectome will then hopefully be further linked and attributed to clinical variables as well (Castellanos et al., 2013), in the framework of the widely spreading connectomic approach (Griffa et al., 2013) and of the future development of recently emerged biological technique (Chung and Deisseroth, 2013; Chung et al., 2013).

There are very good premises to add new chapters in the challenging pursuit of the boundaries of the self in the brain. A work on the self deals with the more intimate meaning of mankind and, quoting the writer Lewis Carroll, will make all of us once again “curiouser and curiouser” toward the wonderland of neuroscience.

## References

[B1] Avenanti A. Urgesi C. (2011). Understanding ‘what’ others do: mirror mechanisms play a crucial role in action perception. Soc. Cogn. Affect. Neurosci. 6, 257–259 10.1093/scan/nsr004 21653637 PMC3110438

[B2] Baumeister R. F. Finkel E. J. (2010). Advanced Social Psychology. The State of the Science. New York: Oxford University Press

[B3] Boly M. Tshibanda L. Vanhaudenhuyse A. Noirhomme Q. Schnakers C. Ledoux D. (2009). Functional connectivity in the default network during resting state is preserved in a vegetative but not in a brain dead patient. Hum. Brain Mapp. 30, 2393–2400 10.1002/hbm.20672 19350563 PMC6870763

[B4] Buckner R. L. (2012). The serendipitous discovery of the brain’s default network. Neuroimage 62, 1137–1145 10.1016/j.neuroimage.2011.10.035 22037421

[B5] Castellanos F. X. Di Martino A. Craddock R. C. Mehta A. D. Milham M. P. (2013). Clinical applications of the functional connectome. Neuroimage [Epub ahead of print]. 10.1016/j.neuroimage.2013.04.083 PMC3809093 23631991

[B6] Castiello U. Becchio C. Zoia S. Nelini C. Sartori L. Blason L. (2010). Wired to be social: the ontogeny of human interaction. PLoS ONE 5:e13199 10.1371/journal.pone.0013199 20949058 PMC2951360

[B7] Cattaneo L. Rizzolatti G. (2009). The mirror neuron system. Arch. Neurol. 66, 557–560 10.1001/archneurol.2009.41 19433654

[B8] Cattaneo L. Sandrini M. Schwarzbach J. (2010). State-dependent TMS reveals a hierarchical representation of observed acts in the temporal, parietal, and premotor cortices. Cereb. Cortex 20, 2252–2258 10.1093/cercor/bhp291 20051360

[B9] Chung K. Deisseroth K. (2013). CLARITY for mapping the nervous system. Nat. Methods 10, 508–513 10.1038/nmeth.2481 23722210

[B10] Chung K. Wallace J. Kim S. Y. Kalyanasundaram S. Andalman A. S. Davidson T. J. (2013). Structural and molecular interrogation of intact biological systems. Nature 497, 332–337 10.1038/nature12107 23575631 PMC4092167

[B11] di Pellegrino G. Fadiga L. Fogassi L. Gallese V. Rizzolatti G. (1992). Understanding motor events: a neurophysiological study. Exp. Brain Res. 91, 176–180 10.1007/BF00230027 1301372

[B12] Enticott P. G. Kennedy H. A. Rinehart N. J. Tonge B. J. Bradshaw J. L. Taffe J. R. (2012). Mirror neuron activity associated with social impairments but not age in autism spectrum disorder. Biol. Psychiatry 71, 427–433 10.1016/j.biopsych.2011.09.001 21974786

[B13] Ferri F. Frassinetti F. Mastrangelo F. Salone A. Ferro F. M. Gallese V. (2012). Bodily self and schizophrenia: the loss of implicit self-body knowledge. Conscious. Cogn. 21, 1365–1374 10.1016/j.concog.2012.05.001 22673373

[B14] Gallagher S. (2011). The Oxford Handbook of the Self. Oxford: Oxford University Press

[B15] Gallese V. Rochat M. J. Berchio C. (2013). The mirror mechanism and its potential role in autism spectrum disorder. Dev. Med. Child Neurol. 55, 15–22 10.1111/j.1469-8749.2012.04398.x 22924341

[B16] Gallup G. G. Jr. (1970). Chimpanzees: self-recognition. Science 167, 86–87 10.1126/science.167.3914.86 4982211

[B17] Gallup G. G. Jr. (1994). “Self-recognition: research strategies and experimental design,” in Self-Awareness in Animals and Humans: Developmental Perspectives, eds Parker S. T. Mitchell R. W. Boccia M. L. (New York: Cambridge University Press), 35–50

[B18] Gallup G. G. Jr. Keenan J. Falk D. (2004). The Face in the Mirror: How We Know Who We Are. New York: Harper Perennial

[B19] Gao W. Zhu H. Giovanello K. S. Smith J. K. Shen D. Gilmore J. H. (2009). Evidence on the emergence of the brain’s default network from 2-week-old to 2-year-old healthy pediatric subjects. Proc. Natl. Acad. Sci. U.S.A. 106, 6790–6795 10.1073/pnas.0811221106 19351894 PMC2672537

[B20] Griffa A. Baumann P. S. Thiran J. P. Hagmann P. (2013). Structural connectomics in brain diseases. Neuroimage [Epub ahead of print]. 10.1016/j.neuroimage.2013.04.056 23623973

[B21] Gusnard D. A. Akbudak E. Shulman G. L. Raichle M. E. (2001). Medial prefrontal cortex and self-referential mental activity: relation to a default mode of brain function. Proc. Natl. Acad. Sci. U.S.A. 98, 4259–4264 10.1073/pnas.071043098 11259662 PMC31213

[B22] Iacoboni M. Dapretto M. (2006). The mirror neuron system and the consequences of its dysfunction. Nat. Rev. Neurosci. 7, 942–951 10.1038/nrn2024 17115076

[B23] Irish M. Piguet O. Hodges J. R. (2012). Self-projection and the default network in frontotemporal dementia. Nat. Rev. Neurol. 8, 152–161 10.1038/nrneurol.2012.11 22331029

[B24] Keysers C. Kaas J. H. Gazzola V. (2010). Somatosensation in social perception. Nat. Rev. Neurosci. 11, 417–428 10.1038/nrn2919 20445542

[B25] Kilner J. M. Blakemore S. J. (2007). How does the mirror neuron system change during development? Dev. Sci. 10, 524–526 10.1111/j.1467-7687.2007.00632.x 17683337

[B26] Kilner J. M. Neal A. Weiskopf N. Friston K. J. Frith C. D. (2009). Evidence of mirror neurons in human inferior frontal gyrus. J. Neurosci. 29, 10153–10159 10.1523/JNEUROSCI.2668-09.2009 19675249 PMC2788150

[B27] Kojima T. Onoe H. Hikosaka K. Tsutsui K. Tsukada H. Watanabe M. (2009). Default mode of brain activity demonstrated by positron emission tomography imaging in awake monkeys: higher rest-related than working memory-related activity in medial cortical areas. J. Neurosci. 29, 14463–14471 10.1523/JNEUROSCI.1786-09.2009 19923280 PMC6665835

[B28] Legrenzi P. Umiltà C. (2011). Neuromania: On the Limits of Brain Science. Oxford: Oxford University Press

[B29] Lepage J. F. Théoret H. (2007). The mirror neuron system: grasping others’ actions from birth? Dev. Sci. 10, 513–523 10.1111/j.1467-7687.2007.00631.x 17683336

[B30] Lingnau A. Gesierich B. Caramazza A. (2009). Asymmetric fMRI adaptation reveals no evidence for mirror neurons in humans. Proc. Natl. Acad. Sci. U.S.A. 106, 9925–9930 10.1073/pnas.0902262106 19497880 PMC2701024

[B31] Lu H. Zou Q. Gu H. Raichle M. E. Stein E. A. Yang Y. (2012). Rat brains also have a default mode network. Proc. Natl. Acad. Sci. U.S.A. 109, 3979–3984 10.1073/pnas.1200506109 22355129 PMC3309754

[B32] Mantini D. Gerits A. Nelissen K. Durand J. B. Joly O. Simone L. (2011). Default mode of brain function in monkeys. J. Neurosci. 31, 12954–12962 10.1523/JNEUROSCI.2318-11.2011 21900574 PMC3686636

[B33] Markus H. R. Kitayama S. (1991). Culture and the self: implications for cognition, emotion, and motivation. Psychol. Rev. 98, 224–253 10.1037/0033-295X.98.2.224

[B34] Martínez Mateo M. Cabanis M. Stenmanns J. Krach S. (2013). Essentializing the binary self: individualism and collectivism in cultural neuroscience. Front. Hum. Neurosci. 7:289 10.3389/fnhum.2013.00289 23801954 PMC3689037

[B35] McCormick L. M. Brumm M. C. Beadle J. N. Paradiso S. Yamada T. Andreasen N. (2012). Mirror neuron function, psychosis, and empathy in schizophrenia. Psychiatry Res. 201, 233–239 10.1016/j.pscychresns.2012.01.004 22510432 PMC3545445

[B36] Mehta U. M. Basavaraju R. Thirthalli J. Gangadhar B. N. (2012). Mirror neuron dysfunction-a neuro-marker for social cognition deficits in drug naïve schizophrenia. Schizophr. Res. 141, 281–283 10.1016/j.schres.2012.07.005 22835807

[B37] Mukamel R. Ekstrom A. D. Kaplan J. Iacoboni M. Fried I. (2010). Single-neuron responses in humans during execution and observation of actions. Curr. Biol. 20, 750–756 10.1016/j.cub.2010.02.045 20381353 PMC2904852

[B38] Northoff G. Duncan N. W. Hayes D. J. (2010). The brain and its resting state activity – experimental and methodological implications. Prog. Neurobiol. 92, 593–600 10.1016/j.pneurobio.2010.09.002 20888388

[B39] Northoff G. Qin P. Feinberg T. E. (2011). Brain imaging of the self – conceptual, anatomical and methodological issues. Conscious. Cogn. 20, 52–63 10.1016/j.concog.2010.09.011 20932778

[B40] Qin P. Northoff G. (2011). How is our self related to midline regions and the default-mode network? Neuroimage 57, 1221–1233 10.1016/j.neuroimage.2011.05.028 21609772

[B41] Raichle M. E. MacLeod A. M. Snyder A. Z. Powers W. J. Gusnard D. A. Shulman G. L. (2001). A default mode of brain function. Proc. Natl. Acad. Sci. U.S.A. 98, 676–682 10.1073/pnas.98.2.676 11209064 PMC14647

[B42] Raichle M. E. Snyder A. Z. (2007). A default mode of brain function: a brief history of an evolving idea. Neuroimage 37, 1083–1090; discussion 1097–1099. 10.1016/j.neuroimage.2007.02.041 17719799

[B43] Rilling J. K. Barks S. K. Parr L. A. Preuss T. M. Faber T. L. Pagnoni G. (2007). A comparison of resting-state brain activity in humans and chimpanzees. Proc. Natl. Acad. Sci. U.S.A. 104, 17146–17151 10.1073/pnas.0705132104 17940032 PMC2040430

[B44] Rizzolatti G. Sinigaglia C. (2010). The functional role of the parieto-frontal mirror circuit: interpretations and misinterpretations. Nat. Rev. Neurosci. 11, 264–274 10.1038/nrn2805 20216547

[B45] Salomon R. Levy D. R. Malach R. (2013). Deconstructing the default: cortical subdivision of the default mode/intrinsic system during self-related processing. Hum. Brain Mapp. 2013 10.1002/hbm.22268 PMC6869590 23568328

[B46] Sandrone S. (2012). The brain as a crystal ball: the predictive potential of default mode network. Front. Hum. Neurosci. 6:261 10.3389/fnhum.2012.00261 23055961 PMC3458239

[B47] Sandrone S. (2013). A DMN-based functional taxonomy of the resting human brain: is essential really invisible to the eye? Brain Res. Bull. [Epub ahead of print]. 10.1016/j.brainresbull.2013.06.008 23838431

[B48] Sheline Y. I. Barch D. M. Price J. L. Rundle M. M. Vaishnavi S. N. Snyder A. Z. (2009). The default mode network and self-referential processes in depression. Proc. Natl. Acad. Sci. U.S.A. 106, 1942–1947 10.1073/pnas.0812686106 19171889 PMC2631078

[B49] Sinigaglia C. Rizzolatti G. (2011). Through the looking glass: self and others. Conscious. Cogn. 20, 64–74 10.1016/j.concog.2010.11.012 21220203

[B50] Uddin L. Q. Iacoboni M. Lange C. Keenan J. P. (2007). The self and social cognition: the role of cortical midline structures and mirror neurons. Trends Cogn. Sci. (Regul. Ed.) 11, 153–157 10.1016/j.tics.2007.01.001 17300981

[B51] van Buuren M. Gladwin T. E. Zandbelt B. B. Kahn R. S. Vink M. (2010). Reduced functional coupling in the default-mode network during self-referential processing. Hum. Brain Mapp. 31, 1117–1127 10.1002/hbm.20920 20108218 PMC6870730

